# A Retrospective Epidemiological Study of Tick-Borne Encephalitis Virus in Patients with Neurological Disorders in Hokkaido, Japan

**DOI:** 10.3390/microorganisms8111672

**Published:** 2020-10-28

**Authors:** Kentaro Yoshii, Ikuko Takahashi-Iwata, Shinichi Shirai, Shintaro Kobayashi, Ichiro Yabe, Hidenao Sasaki

**Affiliations:** 1 Laboratory of Public Health, Faculty of Veterinary Medicine, Hokkaido University, Sapporo 060-0818, Japan; shin-kobayashi@vetmed.hokudai.ac.jp; 2 National Research Center for the Control and Prevention of Infectious Diseases (CCPID), Nagasaki University, Nagasaki 852-8523, Japan; 3 Department of Neurology, Faculty of Medicine and Graduate School of Medicine, Hokkaido University, Sapporo 060-0818, Japan; ikukotak@med.hokudai.ac.jp (I.T.-I.); shirai@med.hokudai.ac.jp (S.S.); yabe@med.hokudai.ac.jp (I.Y.); sasaki-hidenao@hakochu-hp.gr.jp (H.S.)

**Keywords:** tick-borne encephalitis, tick, neurological disorder, epidemiology, zoonosis

## Abstract

Tick-borne encephalitis (TBE) is a zoonotic disease that usually presents as a moderate febrile illness followed by severe encephalitis, and various neurological symptoms are observed depending on the distinct central nervous system (CNS) regions affected by the TBE virus (TBEV) infection. In Japan, TBE incidence is increasing and TBEV distributions are reported in wide areas, specifically in Hokkaido. However, an extensive epidemiological survey regarding TBEV has not been conducted yet. In this study, we conducted a retrospective study of the prevalence of antibodies against TBEV in patients with neurological disorders and healthy populations in a TBEV-endemic area in Hokkaido. Among 2000 patients, three patients with inflammatory diseases in the CNS had TBEV-specific IgM antibodies and neutralizing antibodies. The other four patients diagnosed clinically with other neurological diseases were positive for TBEV-specific IgG and neutralizing antibodies, indicating previous TBEV infection. In a total of 246 healthy residents in a TBEV-endemic region, one resident had TBEV-specific antibodies. These results demonstrated undiagnosed TBEV infections in Japan. Further surveys are required to reveal the actual epidemiological risk of TBE and to consider preventive measures, such as a vaccine program, for the control of TBE in Japan.

## 1. Introduction

Tick-borne encephalitis (TBE) is a zoonotic disease that usually presents as a moderate febrile illness followed by severe encephalitis. TBE is caused by TBE virus (TBEV), a single-stranded, positive-sense RNA virus of the genus *Flavivirus*. TBEV is transmitted by tick bites and is maintained in the zoonotic transmission cycle between ticks and wild vertebrate hosts. TBEV is prevalent over a wide area of the Eurasian continent, including Europe, Russia, and Far-Eastern Asia, including Japan [1,2,3], and more than 10,000 patients with TBE are reported annually. Based on phylogenetic analysis, TBEV can be divided into at least three subtypes: the Far-Eastern subtype, known as the Russian spring-summer encephalitis virus, the European subtype, and the Siberian subtype [2,4]; the Baikalian and Himalayan subtypes have also been recognized recently [5,6,7].

After 7–10 days of incubation, TBE symptoms appear in a two-phase course but they vary in the subtypes. Approximately 30% of infected individuals remain asymptomatic. Flu-like symptoms are observed during the initial viremic phase of the illness, which include fever and headache [8,9]. After TBEV invades the brain, various neurological symptoms are observed in the second phase. A biphasic course is observed in TBE patients infected with the European subtype, while infection with the Siberian and Far-Eastern subtypes of TBEV are predominantly monophasic (i.e., absence of the initial viremic phase). Altered mental state is the most common neurological symptom. Disorientation, excitation, seizures, and confusion and cerebellar signs, depending on distinct central nervous system (CNS) regions affected by TBEV infection, are also observed. These symptoms are difficult to differentiate from those of other CNS diseases. Thus, laboratory confirmation is necessary for a definitive diagnosis but it is significantly limited in Japan.

In Japan, two patients infected with a virus within the TBE serocomplex had encephalitis during an epidemic of Japanese encephalitis (JE) in 1948 in the Tokyo area. The isolated virus, named Negishi virus, was retrospectively identified as a member of the louping ill virus through phylogenetic analyses performed decades later [10,11]. No subsequent cases of TBE in Japan were reported until 1993, when a patient with viral encephalitis in southern Hokkaido, the northern island of Japan, was diagnosed with TBE [12]. Since this first confirmed TBE case in 1993, only four additional cases of TBE were reported in a wide area of Hokkaido in Japan between 2016 and 2018 [13,14]. The Far-Eastern subtype of TBEV was isolated from dogs, wild rodents, and *Ixodes ovatus* ticks in an area where the TBE patients were reported [15,16,17,18,19]. In epizootiological surveys in Japan, high seropositivity rates (approximately 10% to 20%) against TBEV were detected in wild rodents in several cities or towns in southern Hokkaido, and seropositive animals such as dogs, horses and deer were sporadically detected in Hokkaido [18,20,21]. In our sero-epidemiological studies in humans, a meningoencephalitis patient suspected of having Lyme disease was found to be infected with TBEV, and unrecognized subclinical infections of TBEV were detected among members of the Japan Self-Defense Force [22,23]. These findings indicated the possibility of overlooked TBE cases and asymptomatic infections. To control TBE in Japan, it is necessary to determine the actual endemic situation, but an extensive epidemiological survey regarding TBEV has not been conducted.

Here, we conducted a retrospective study of the prevalence of antibodies against TBEV in patients with neurological disorders who visited the Department of Neurology at Hokkaido University Hospital and the healthy population in a TBEV-endemic area in Hokkaido to investigate the epidemiological risk of TBEV endemic in this part of Japan.

## 2. Materials and Methods

### 2.1. Samples from Subjects

Blood and cerebrospinal fluid (CF) samples were collected from patients with neurological disorders, and blood samples from healthy volunteers in Yuni town between 2010 and 2018 were collected to identify previously undiagnosed TBE infections. Approval for this study was obtained from the Medical Ethics Committee of the Faculty of Veterinary Medicine, Hokkaido University Hospital (research No. 017-0538, 13 June 2018). Written informed consent was obtained from all participants.

### 2.2. Detection of Anti-TBEV Specific Antibodies

#### 2.2.1. IgG-enzyme-Linked Immunosorbent Assay (ELISA) Using Subviral Particles (SPs)

Anti-TBEV IgG antibodies were examined by SP-IgG-ELISA, previously developed in [24]. Briefly, human embryonic kidney 293T (HEK293T) cells were transfected with the pCAG-TBEV-M-StrepE plasmid expressing subviral particles (SPs) tagged with Strep-tag. These cells were cultured in Dulbecco’s Modified Eagle Medium supplemented with 4.5 g/L d-glucose (FUJIFILM, Osaka, Japan) and 10% fetal bovine serum (FBS) at 37 °C. The SPs in the supernatant were precipitated with final concentrations of 10% polyethylene glycol 8,000 and 1.9% sodium chloride and used as antigens for ELISA. Moreover, 96-well EIA plates (Corning, New York, NY, USA) were coated with Strep-Tactin overnight at 4 °C and subsequently blocked with Block Ace (DS Pharma Biomedical, Osaka, Japan). After washing with PBS containing 0.05% Tween 20, the antigen SPs were added, followed by serum samples. The TBEV-specific antibodies were detected by Protein A/G conjugated with horseradish peroxidase (HRP) (Thermo Fisher Scientific, Waltham, MA, USA) and reacted with o-phenylenegiamine dihydrochloride (OPD) in the presence of 0.07% hydrogen peroxide (H_2_O_2_). Negative control antigens were prepared from the supernatant of untransfected HEK293T cells. The results were recorded as the P/N ratio (optical density value using the SPs to that using negative control antigen), and a cutoff value of 1.26 was used.

#### 2.2.2. IgM-ELISA Using SPs

Anti-TBEV IgM antibodies were examined by SP-IgM-ELISA, previously developed in [25]. Briefly, IgM antibodies were captured by anti-human IgM antibody (Bethyl Laboratories, Inc., Montgomery, TX, USA) on 96-well plates after blocking with a Block Ace. The antigen SPs, prepared as described above, were added and detected by Strep-Tactin conjugated with HRP (Bio-Rad, Hercules, CA, USA). The color reaction was developed by OPD in the presence of 0.07% H_2_O_2_. The results were recorded as the P/N ratio, and a cutoff value of 1.30 was used.

#### 2.2.3. Neutralization Test (NT).

TBEV Oshima 5–10 strain [16] was incubated with serially diluted serum and inoculated to baby hamster kidney (BHK) cells. The cells were incubated with Eagle’s Minimal Essential Medium (FUJIFILM, Osaka, Japan) containing 1.5% carboxymethyl cellulose and 2% FBS for 4 days. After 4 days of incubation, the cells were fixed with 10% formalin and stained with 0.1% crystal violet. Serum samples that produced a 50% reduction in plaque formation of the TBEV on BHK cells in 12-well plates were determined and serum samples ≥ 1:10 were considered to be positive for neutralizing antibodies against TBEV.

#### 2.2.4. Interpretation of Serological Results

Serum samples were first screened by IgG- and IgM-ELISA using SPs of TBEV. Sero-positive samples were examined by neutralization tests (NTs) against TBEV and JE virus (JEV) for further confirmation. The samples showing anti-TBEV neutralizing titer ≥ 20 and no anti-JEV neutralizing titer, or at least 4-times higher neutralizing titer against TBEV than JEV, were defined as TBEV infection. The serologic constellations were interpreted as described previously [26]: IgM (+) and IgG (−), early phase of infection; IgM (−) and IgG (+), past infection or vaccination; IgM (+) and IgG (+), acute infection.

### 2.3. RT-PCR and Isolation of TBEV

CF samples suspected of recent TBEV infection were subjected to RT-PCR to detect TBEV genomic RNA. Total RNA was extracted using ISOGEN II (Nippon Gene, Tokyo, Japan) and reverse-transcribed using random primers and Superscript III reverse-transcriptase (ThermoFisher Scientific, Waltham, MA, USA). TBEV-specific sequences were amplified using Platinum Taq polymerase (Thermo Fisher Scientific, Waltham, MA, USA). To amplify the envelope (E) protein gene of TBEV, we used the following primers specific for Far-Eastern TBEV: (forward) 5′-AGATTTTCTTGCACGTGCAT-3′ and (reverse) 5′-GCACACTGT1GTATGTAAGAC-3′.

In order to isolate TBEV from the CF samples, they were inoculated into the BHK cells and incubated at 37 °C under 5% CO_2_. After 2–4 days, the cells were inspected for cytopathic effects (CPEs) and total RNA was extracted for RT-PCR.

## 3. Results

### 3.1. Anti-TBEV Antibodies in Patients with Neurological Disorders in Hokkaido

To investigate unconfirmed TBEV infection, we investigated anti-TBEV antibodies in 2000 patients with neurological disorders who visited the Department of Neurology in Hokkaido University Hospital from 2010 to 2018. The breakdown information of the patients is listed in Table 1. The serum samples were first subjected to IgG- and IgM-ELISA using SPs of TBEV [24,25], and IgG- or IgM-positive samples were subjected to NT for TBEV and JEV. In Japan, JEV, which belongs to mosquito-borne flaviviruses, is widely endemic, and many inhabitants from the main islands of Japan are vaccinated against JEV during their childhood. To examine cross-reactivity against antibodies to other flaviviruses [27,28], the NT titers were compared between TBEV and JEV.

As shown in Table 2, nine and five samples were positive by IgG- and IgM-ELISA, respectively. One sample was positive by both IgG- and IgM-ELISA and neutralizing antibodies against TBEV were also confirmed. Eight samples were positive by IgG-ELISA but negative by IgM-ELISA. Among them, neutralizing antibodies against TBEV were confirmed in seven samples. Four samples were positive for IgM-ELISA but not by IgG-ELISA. Among them, neutralizing antibodies against TBEV were confirmed in three samples. The other 1987 samples were negative by both IgG- and IgM-ELISA.

Detailed information of the antibody-positive samples is shown in Table 3. The three IgM-positive samples had significantly higher titers of neutralizing antibodies against TBEV than JEV, indicating TBE infection. Four IgG-positive but IgM-negative samples had significantly higher titers of neutralizing antibodies against TBEV than JEV, indicating TBE infection, but the neutralizing titers against TBEV were not significantly high (NT_50_ = 20). Three IgG-positive but IgM-negative samples had neutralizing antibodies against TBEV, but significant differences from neutralizing titers against JEV were not observed. One IgG-negative but IgM-positive sample had a low titer of neutralizing antibody against TBEV (NT_50_ = 10), but no significant difference was observed from that against JEV. One IgG-positive but IgM-negative sample and one IgG-negative but IgM positive sample were negative in the NT against both TBEV and JEV.

Detection of TBEV RNA and isolation of TBEV were performed using the CF samples with TBEV-specific IgM and neutralizing antibodies. However, TBEV RNA was not detected in the CF samples. BHK cells inoculated with the CF samples did not show any CPE and no TBEV RNA was detected in the cells.

### 3.2. Sero-epidemiological Survey of Residents in a TBEV-endemic Area

To investigate the natural infection rate in residents in a TBEV-endemic area, a serological survey was conducted in healthy volunteers in Yuni town in central Hokkaido, where TBEV antibodies were detected in wild animals. In total, 246 serum samples were collected and subjected to IgG-ELISA using SPs of TBEV, followed by NT against TBEV. One sample from an 80-year-old woman was positive for IgG and neutralizing antibody against TBEV.

## 4. Discussion

In this study, we conducted a large sero-epidemiological survey of TBEV infection in patients with neurological disorders and a healthy population in a TBEV-endemic area in Hokkaido, Japan. For the first screening of the subjects, we conducted IgG- and IgM-ELISA using SPs, which had higher specificity and sensitivity with minimum cross-reactivity with other flavivirus infections compared to the commercial ELISA using formalin-inactivated virion [24,25]. ELISA-positive samples were confirmed by NT, and negative results in NTs were interpreted as false-positive results in ELISAs. As none of the IgM- and/or IgG-positive samples showed higher neutralizing titers against JEV than TBEV, no apparent cross-reactivity by anti-JEV antibodies was observed by our ELISA using SPs.

The positive samples by serological assays were speculated according to Dr. Dobler’s previous interpretation of serologic constellations [26] (Table 3). The three IgM-positive samples with significant anti-TBEV neutralizing antibodies were considered to be in the early phase of TBEV infection. One of them also had IgG antibodies, but the possibility of the effect of previous TBEV vaccination could be excluded because there was no history of the vaccine. All three patients showed inflammatory disease in the CNS, which might be caused by TBEV infection.

The other ten IgG- or IgM-positive samples were clinically diagnosed as other neurological diseases, not inflammatory diseases. Four of them had significantly higher titers of neutralizing antibodies against TBEV than JEV. In Japan, JEV is widely endemic and JE cases have been reported almost annually. However, no JE cases were reported in Hokkaido due to the limited distribution of mosquito vectors (*Culex tritaeniorhynchus*), and routine vaccination against JEV was not conducted until 2018. Therefore, many residents in Hokkaido do not possess JEV antibodies. Taking into consideration these regional situations, these subjects were considered to have previous TBEV infection.

A serological survey in patients with a CNS infection between 1997 and 2012 was conducted in Finland and 0.25% (5/1957) were found to be positive [29]. The positive ratio was relatively similar to our results in which 0.37% (3/819) were positive in patients with inflammatory disease. These results indicated that undiagnosed TBE existed in Hokkaido, Japan, although it forms a minor fraction of the patient group. In Finland, increased incidence of TBE was reported in the residents of the Helsinki area in concordance with observations on the changing epidemiology [30]. A similar situation can be considered in Hokkaido and further epidemiological surveys are required.

In Japan, no epidemiological survey has been conducted in the residents of TBEV-endemic regions. Our survey detected one person with the TBEV-specific antibody out of the 246 individuals included (0.4%). This seroprevalence rate was relatively lower than the usual ratio reported in studies in European countries (0–5%) [31,32,33,34,35]. Previous studies in high-risk populations who have a high frequency of tick-bite during work also showed a lower seroprevalence rate in Hokkaido (0.7%) than that conducted in European countries (3–16%) [31,35]. These data suggest that the opportunity for TBEV infection might not be significantly high in Japan. Actually, a survey of ticks in TBEV-endemic areas in Hokkaido showed relatively lower minimum field detection rates of TBEV in *Ixodes ovatus* (0.05–0.33%) as compared to surveys in other endemic countries [17,19]. These low infection rates might be due to poor vector competency of *I. ovatus* because *I. persulcatus* is the primary vector of TBEV in Far-Eastern Russia and Asia.

In conclusion, our surveillance revealed undiagnosed TBE in patients with neurological disorders and previous TBEV infection in an endemic region in Hokkaido, Japan, for the first time. The incidence of TBE in Japan is increasing, and sero-epizootiological evidence for TBEV distribution has been reported not only in a wide area of Hokkaido but also in the main islands of Japan. However, awareness of TBE is significantly low, even in physicians, and only limited facilities can conduct laboratory diagnosis of TBEV infection in Japan. No vaccine against TBEV has been licensed in Japan. There is an urgent need to reveal the actual epidemiological risk of TBE and to consider preventive measures, such as a vaccine program, for the control of TBE in Japan.

## Figures and Tables

**Table 1:** Breakdown of information of the patients.

		No.	Percentage
Gender	Female	1041	52.1%
	Male	959	48.0%
Age	10–19	49	2.5%
	20–29	143	7.2%
	30–39	171	8.6%
	40–49	231	11.6%
	50–59	295	14.8%
	60–69	508	25.4%
	70–79	467	23.4%
	80–89	129	6.5%
	90–99	7	0.4%
Classification of diseases	Neurodegenerative disease	819	41.0%
	Autoimmune disease	283	14.2%
	Inflammatory disease	245	12.3%
	Peripheral neuropathy	237	11.9%
	Muscle disease	81	4.1%
	Neoplastic disease	61	3.1%
	Metabolic disease	54	2.7%
	Vascular disorder	43	2.2%
	Neuromuscular junction disorder	34	1.7%
	Infectious disease (without inflammatory disease)	32	1.6%
	Spinal disease	28	1.4%
	Cerebrospinal fluid circulation disorder	27	1.4%
	Functional disease	26	1.3%
	Mental disease	15	0.8%
	Others	15	0.8%

**Table 2:** Summary of serological tests for anti-TBEV antibodies.

		IgG		Total
		Positive	Negative	
IgM	Positive	1 (1) ^1^	4 (3)	5
	Negative	8 (7)	1987 (NT) ^2^	1995
Total		9	1991	2000

^1^ Number of samples which showed neutralization against tick-borne encephalitis virus (TBEV). ^2^ Not tested by neutralization test.

**Table 3:** Details of anti-TBEV antibodies in sero-positive subjects.

Age	Gender	Classification of Diseases	IgG	IgM	TBEV NT	TBEV JE
70	Female	Inflammatory disease	(+)	(+)	>160	40
25	Male	Inflammatory disease	(−)	(+)	160	20
57	Male	Inflammatory disease	(−)	(+)	40	<10
55	Female	Autoimmune disease	(+)	(−)	20	<10
70	Female	Neoplastic disease	(+)	(−)	20	<10
64	Female	Neurodegenerative disease	(+)	(−)	20	<10
25	Female	Peripheral neuropathy	(+)	(−)	20	<10
81	Female	Infectious disease (without inflammatory disease)	(+)	(−)	20	10
60	Female	Vascular disorder	(+)	(−)	10	<10
68	Female	Neurodegenerative disease	(+)	(−)	10	<10
62	Male	Functional disease	(−)	(+)	10	<10
33	Male	Others	(+)	(−)	<10	<10
70	Male	Autoimmune disease	(−)	(+)	<10	<10
